# Emerging 2D Nanomaterials for Multimodel Theranostics of Cancer

**DOI:** 10.3389/fbioe.2021.769178

**Published:** 2021-11-19

**Authors:** Wei Zhu, Helin Li, Peng Luo

**Affiliations:** ^1^ Key Laboratory of Advanced Textile Materials and Manufacturing Technology and Engineering Research Center for Eco-Dyeing and Finishing of Textiles, Ministry of Education, Zhejiang Sci-Tech University, Hangzhou, China; ^2^ Collaborative Innovation Center of Yangtze River Delta Region Green Pharmaceuticals, Zhejiang University of Technology, Hangzhou, China; ^3^ Institute of Technical and Macromolecular Chemistry, RWTH Aachen University, Aachen, Germany; ^4^ Department of Orthopedic Trauma, The Second Affiliated Hospital and Yuying Children’s Hospital of Wenzhou Medical University, Wenzhou, China

**Keywords:** 2D nanomaterials, nanomedicine, theranostics, clinical, cancer

## Introduction

**FIGURE 1:**
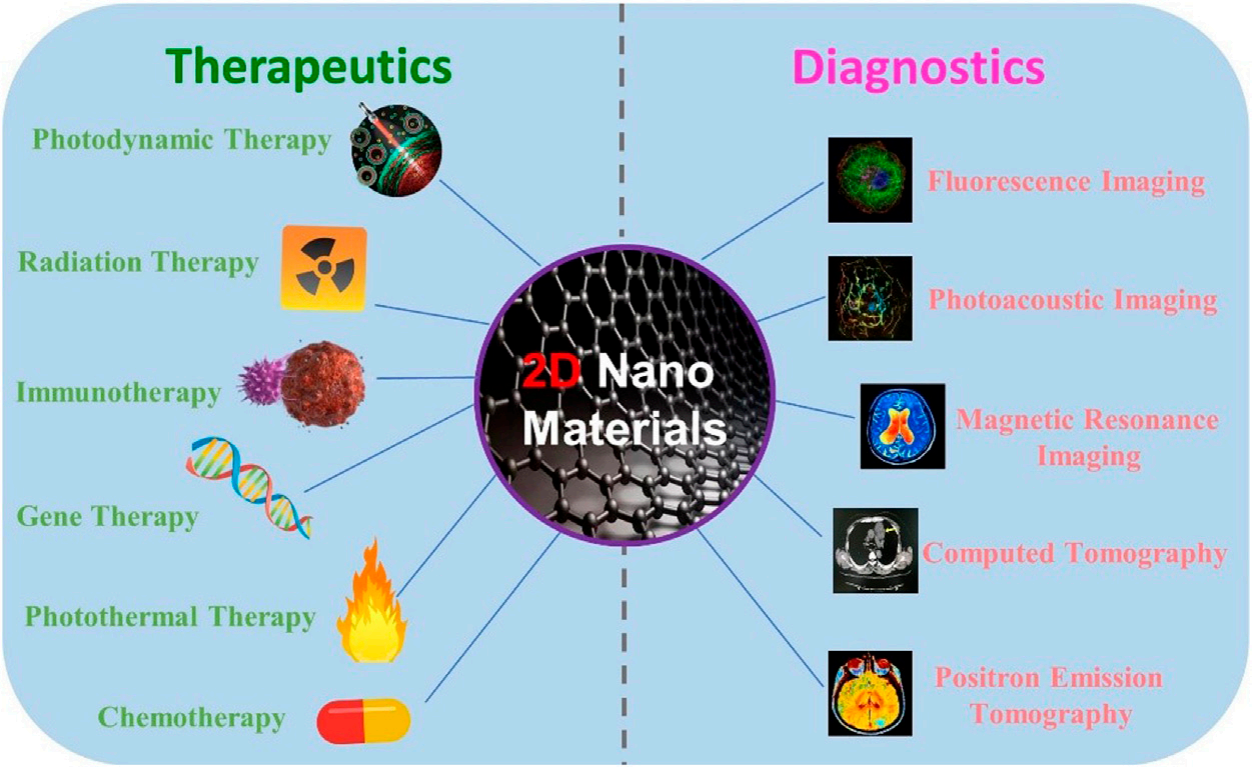
Treatment and diagnosis methods based on the combined 2D nanomaterials. Some elements are adapted with permissions from Wang et al. (2018), copyright 2018 Ivyspring International Publisher.

Cancer is a major public health problem worldwide nowadays, with more than 18 million new cases each year. In 2020, the diagnosis and treatment of cancer were interfered by the coronavirus disease 2019 (COVID-19) pandemic. Reduced access to care resulted in delays in diagnosis and treatment in relation to increased death (Li et al., 2021b; Siegel et al., 2021). Although cancer treatment strategies were developed, it is still extremely important to speed up the diagnosis and treatment of cancer. Recently, theranostics have stimulated increased attention in both research and clinical fields, which allow very intelligent diagnostic imaging ability with therapeutic intervention within spatial colocalization (Li et al., 2017b; Kang et al., 2020; Li et al., 2020). Up to now, various theranostic systems have been explored, involving different modalities of diagnosis and therapies. To gain versatility, increasingly complex nanoparticles are designed to enable multimodal imaging and combination therapy (Li et al., 2017a; Chen et al., 2018). However, the purpose brings the difficulty of nanomaterials with a great deal of uncertainty, which seriously hampers clinical progress. For clinical transformation, the key is to achieve image-mediated therapy with the simpler components of nanomaterials (Li H. et al., 2019; Li et al., 2019b; Li et al., 2021c; Li et al., 2021d). Over the last few years, two-dimensional (2D) nanomaterials have been widely used for cancer diagnosis and treatment with the design based on simple components (Figure 1) (Wang and Cheng, 2019; Cheng et al., 2020; Wang et al., 2021).

Compared with other nanomaterials, e.g., liposome, dendrimer, and carbon nanotube, 2D nanomaterials have unique advantages that enable them to be requested as a biomedicine so conveniently (Gazzi et al., 2019; Gravagnuolo et al., 2021). Firstly, the rich source of 2D nanomaterials provides plentiful resources to meet different requirements for applications, including hexagonal boron nitride, group-VA semiconductors, graphitic carbon nitride, transition metal carbides, and transition metal dichalcogenides. Second, the good chemical, physical, and biological properties of 2D nanomaterials, such as optical, magnetic, electrical, or catalytic properties, can be well matched to provide desirable performance for diagnostics, imaging, or therapy of cancer that can be applied in the fields of practical biomedical applications. Third, the preparation of 2D nanomaterials is relatively simple with good yields in the laboratory (Zhang et al., 2021). The feasibility of 2D nanomaterials points out that they can be developed as promising clinical nanoplatforms for cancer theranostics (Huang et al., 2021).

Herein, the recent processes of the synthesis and applications of 2D nanomaterials for the treatment and diagnosis of cancer were discussed and summarized. Based on the large surface area and exceptional physicochemical properties, the various kinds of 2D nanomaterials were developed in the field of nanomedicine due to their excellent multimodal-imaging-guided synergetic cancer therapy performance. Moreover, 2D nanomaterials also can be developed in the field of cancer immunotherapy through encapsulating bioinspired cell membranes for cancer-targeting therapy and, thus, provides an advance in personalized immunotherapy. Encouragingly, a safe and efficient 2D nanomaterial platform has been reported to realize the clinical nanomedicines with excellent efficacy of survival rate of 100% *in vivo* without preparing the complex nanoplatforms.

## Discussion

Owing to the large surface area and exceptional physicochemical properties of 2D nanomaterials, 2D nanomaterial-based multifunctional nanocomposites are promising materials for multimodal-imaging-guided synergetic cancer therapy (Table 1) (Zhong et al., 2021). A lot of theranostic platforms have been developed, involving diagnosis [computed tomography (CT), fluorescence imaging (FI), magnetic resonance imaging (MRI), photoacoustic imaging (PAI), positron emission tomography (PET), etc.] and therapies [chemotherapy (CHT), photothermal therapy (PTT), photodynamic therapy (PDT), radiation therapy (RT), gene therapy (GT), immunotherapy (IT), etc.] (Li X. et al., 2014; Li et al., 2016; Xing et al., 2020). Multimodal is far superior to single-component imaging and diagnosis, which are complementary and enhance each other. Taking triple-modal PAI/MRI/CT as examples, the strong near-infrared (NIR) absorbance of WS_2_ with high photothermal conversion efficiency enables PA imaging; the WS_2_ nanosheet-doped Gd^3+^ ions offer a strong contrast in T1-weighted MR imaging. Meanwhile, W and Gd elements could attenuate X-ray irradiation to allow for CT imaging (Li X. et al., 2014). Graphene is a 2D layer of carbon atoms that can be used in a wide range of applications including nanomedicine (Tufano et al., 2020). Owing to its extremely large specific surface areas, graphene has great potential in biosensors, drug delivery, and cancer treatment (Gu et al., 2019). Bianco and co-workers recently introduced a multifunctional drug delivery platform based on graphene for cancer therapy applications (Lucherelli et al., 2020). The multifunctional graphene platform, modified with indocyanine green as the fluorophore, folic acid as the targeting agent to Hela cells, and doxorubicin (DOX) as the therapeutic molecule, showed a combined synergistic effect of targeting drug release of DOX for selectively killing cancer and photothermal properties under NIR irradiation. A significant reduction of Hela cell viability was observed, suggesting that the nanoplatform has been proven for effective anticancer therapy attributed to the synergistic effect of chemo- and photothermal therapies. Moreover, due to its good biocompatibility and biodegradability, black phosphorus nanoparticles have attracted more and more attention in the biomedical field in recent years (Zhang et al., 2021). Tang and his colleagues demonstrated a facile method to construct a new aggregation-induced emission (AIE) photosensitizer combined with 2D black phosphorus nanosheets and their application for multimodal theranostics involving NIR fluorescence–photothermal dual imaging-guided synergistic photodynamic–photothermal therapy (Huang et al., 2020). With high stability and good biocompatibility, the hybrid nanomaterial can effectively generate reactive oxygen species and show bright NIR fluorescence and excellent photothermal conversion efficiency. It also exhibits the effective lysosomal escape and mitochondria targeting effects due to the amine groups that protonated at the acidic tumor microenvironment. These remarkable characteristics make it have enhanced antitumor efficacy to 4T1 skin tumor. In recent years, cancer immunotherapy has begun to attract widespread attention, becoming an effective method in the clinical treatment of cancer. Through encapsulation with cell membranes, 2D materials have become popular in cancer immunotherapy that can be used as a biomimetic nanocarrier to load anticancer drugs for cancer-targeting therapy. Chen and his colleagues reported that bioinspired red blood cell (RBC) membrane is used for wrapping 2D MoSe_2_ nanosheets with high photothermal conversion efficiency to achieve enhanced biocompatibility and circulation time (He et al., 2019). 2D MoSe_2_ nanosheets encapsulated with cell membranes has tumor-targeting capability. The combination of RBC–MoSe_2_ with anti-PD-1 immunotherapy prevented the activation of the PD-1/PD-L1 pathway to avoid immune failure and stopped the transmission of an antiapoptotic signal to tumor cells, indicating the specific immune responses to CT 26 colorectal tumor. This RBC–MoSe_2_-potentiated PTT demonstrated the efficient photothermal-potentiated systemic cancer immunotherapy via utilizing biomimetic 2D nanomaterial that was effectively able to kill cancer cells and, thus, provides potential advance for clinical translation.

**TABLE 1:** Classification and applications of 2D material used for multimodel theranostics of cancer.

2D material type	Treatment means	Imaging method	Cancer type	References
GO^a^	CHT, PTT	FI	Lymph cancer	Sun et al. (2008)
GO	CHT, GT, PTT	FI, PET, CT	Breast cancer	Yang et al. (2013)
GO	PDT, PTT	FI	Oral cancer	Tian et al. (2011)
GO	RT, PTT	CT, X-ray	Breast cancer	Chen et al. (2015)
GO	IT, PTT	FI	Colon cancer	Yan et al. (2019)
TMDs^b^ (ReS_2_)	RT, PTT	CT, PAI	Breast cancer	Qian et al. (2015)
TMDs (MoS_2_)	CHT, PTT	FI	Breast cancer	Liu et al. (2014)
TMDs (MoS_2_)	GT, PTT	FI	Rectal cancer	Kim et al. (2016)
TMDs (MoS_2_)	IT, PTT	FI	Leukemia	Han et al. (2017)
TMDs (WS_2_)	PTT, RT	PAI, CT, MRI	Breast cancer	Cheng et al. (2015)
Mxenes (Ti_3_C_2_)	PTT	FI	Breast cancer	Lin et al. (2017b)
Mxenes (Ta_4_C_3_)	PTT	PAI, CT	Breast cancer	Lin et al. (2018)
Mxenes (Nb_2_C)	PTT	PAI	Breast cancer	Lin et al. (2017a)
BP^c^	PDT	FI	Cervical cancer	Lv et al. (2016)
BP	CHT	FI	Cervical cancer	Tao et al. (2017)
LDHs^d^	CHT, PTT, PDT	FI	Liver cancer	Peng et al. (2018)
LDHs	CHT, GT	FI	Breast cancer	Li et al. (2014a)
2D MOF^e^	PDT, PTT	MRI	Osteosarcoma	Li et al. (2018)
hBN^f^	CHT	FI	Prostate cancer	Weng et al. (2014)

a GO, graphene oxide.

b TMDs, transition metal dichalcogenides.

c BP, black phosphorous.

d LDHs, layered double hydroxides.

e MOF, metal–organic frameworks.

f hBN, hexagonal boron nitride.

Despite many studies of 2D nanomaterials used in theranostics of cancer, cases of conversion to the clinic are rarely reported. The recent article by Xing and fellow workers is both timely and exciting for 2D nanomaterial clinical translation (Li et al., 2021a). In this study, the α-tocopherol succinate (α-TOS)-modified two-dimensional molybdenum disulfide (MoS_2_) platform was successfully developed for collaborative computed tomography (CT)/photoacoustic (PA)/photothermal imaging and selective chemotherapy of ovarian cancer. First, the platform has a safe irradiation dose, and its photothermal efficiency (65.3%) is much higher than that of other photothermal materials (ICG = 3.1%, cyanine dyes = 26.6%, and gold nanorods = 21.0%) (Jung et al., 2017). Moreover, the α-TOS is introduced to the platform through a covalent link to realize the selective chemotherapy of cancer cells. The targeted ligand FA is used for specific targeting to achieve effective accumulation in tumor. Owing to good properties, the platform can completely cure solid tumors through photothermal therapy and then kill the remaining cancer cells by selective chemotherapy. The photothermal-selective chemotherapy platform exhibits a synergistic effect in tumor treatment. Moreover, the platform, as a control agent of cooperative CT/PA/thermal images, is useful to achieve precise localization of tumor before performing combined therapy. Crucially, there were almost no side effects during the whole treatment. Its good efficacy and safety *in vivo* make mice survival rate reach 100% in 91 days. Remarkably, the platform can be biodegraded and metabolized *in vivo*. According to these latest clinical transformation concepts, α-TOS combines 2D MoS_2_ as a promising treatment platform, which can be used to achieve convincing efficacy and safety benefits of cancer treatment.

## Conclusion

In conclusion, due to their unique physical and chemical properties, 2D nanomaterials can be used as a platform to realize highly integrated imaging and treatment functions for various types of cancer. We presented the recent progress of the fabrication and studies of 2D nanomaterials, with particular attention on the viewpoints of multimodal-imaging-guided synergetic cancer therapy and cancer immunotherapy. However, despite the reported exciting results, future clinical application of 2D nanomaterials still faces great challenges such as toxicity, low yield, and difficulties in clinical transition. In terms of clinical transformation, the main obstacle is the potential long-term safety of these nanomaterials, especially those nonbiodegradable nanomaterials that remain in the body for a long time. For future clinical application of 2D nanomaterials in the medical field, the following six aspects should be focused on: 1) potential untargeted toxicity from the material, which requires more systematic clinical testing; 2) selection of specific functional materials prior to specific types of cancer treatment; 3) functional optimization of materials; 4) the ongoing concern for the degradability of materials; 5) the imbalance between increasing nanomedicines and low clinical translation; and 6) a more biocompatible and biosafe nanoplatform. Although there are still many knowledge gaps in the field, virtuous perspectives for 2D nanomaterials were evidenced by remarkable progress in recent years. Therefore, 2D nanomaterials, especially those biodegradable nanomaterials, may indeed be a promising application of nanomedical systems in cancer treatment.
